# RETRACTION: Profiling of Circulating Tumor Cells for Screening of Selective Inhibitors of Tumor‐Initiating Stem‐Like Cells

**DOI:** 10.1002/advs.202505952

**Published:** 2025-04-11

**RETRACTION**: C.‐L. Chen, J. C. Hernandez, D. B. U. Kumar, T. Machida, S. M. Tahara, A. El‐Khoueiry, M. Li, V. Punj, S. K. Swaminathan, A. Kirtane, Y. Chen, J. Panyam and K. Machida “Profiling of Circulating Tumor Cells for Screening of Selective Inhibitors of Tumor‐Initiating Stem‐Like Cells,” *Advanced Science* 10, no. 14 (2023): 2206812, https://doi.org/10.1002/advs.202206812.

The above article, published online on 22 March 2023 in Wiley Online Library (wileyonlinelibrary.com), has been retracted by agreement between the authors; the journal Editor‐in‐Chief, Kirsten Severing; and Wiley‐VCH GmbH. The retraction has been agreed upon due to duplication between the AKT1 bands in Figure 5B and the β actin bands in Figure 5D. The β actin bands in Figure 5I,K of this article are also duplicated. Furthermore, elements of Figures 3I,K and 5I have been published in other articles by some of the same authors; in some cases, the duplicated elements are used to represent different scientific contexts. The authors provided an explanation and some data; however, this was not considered satisfactory. Due to the extent and nature of the duplications, the editors consider the results and conclusions substantially compromised.

